# Supercritical Fluid Extraction of *Eucalyptus globulus* Bark—A Promising Approach for Triterpenoid Production

**DOI:** 10.3390/ijms13067648

**Published:** 2012-06-21

**Authors:** Rui M. A. Domingues, Eduardo L. G. Oliveira, Carmen S. R. Freire, Ricardo M. Couto, Pedro C. Simões, Carlos P. Neto, Armando J. D. Silvestre, Carlos M. Silva

**Affiliations:** 1 CICECO, Department of Chemistry, University of Aveiro, Aveiro, 3810-193, Portugal; E-Mails: rdomingues@ua.pt (R.M.A.D.); elgo@ua.pt (E.L.G.O.); cfreire@ua.pt (C.S.R.F.); cneto@ua.pt (C.P.N.); armsil@ua.pt (A.J.D.S.); 2 REQUIMTE, Department of Chemistry, Faculty of Science and Technology, New University of Lisbon, Caparica, 2829-516, Portugal; E-Mails: ricardo.couto@dq.fct.unl.pt (R.M.C.); pcs@dq.fct.unl.pt (P.C.S.)

**Keywords:** *Eucalyptus globulus*, bark, triterpenoids, triterpenic acids, supercritical fluid extraction, carbon dioxide, biorefinery

## Abstract

Eucalyptus bark contains significant amounts of triterpenoids with demonstrated bioactivity, namely triterpenic acids and their acetyl derivatives (ursolic, betulinic, oleanolic, betulonic, 3-acetylursolic, and 3-acetyloleanolic acids). In this work, the supercritical fluid extraction (SFE) of *Eucalyptus globulus* deciduous bark was carried out with pure and modified carbon dioxide to recover this fraction, and the results were compared with those obtained by Soxhlet extraction with dichloromethane. The effects of pressure (100–200 bar), co-solvent (ethanol) content (0, 5 and 8% wt), and multistep operation were studied in order to evaluate the applicability of SFE for their selective and efficient production. The individual extraction curves of the main families of compounds were measured, and the extracts analyzed by GC-MS. Results pointed out the influence of pressure and the important role played by the co-solvent. Ethanol can be used with advantage, since its effect is more important than increasing pressure by several tens of bar. At 160 bar and 40 °C, the introduction of 8% (wt) of ethanol greatly improves the yield of triterpenoids more than threefold.

## 1. Introduction

Presently, technologies based on the use of fossil fuels for energy and chemical production are still predominant. Nonetheless, because of dwindling feedstocks, growing concerns about global climate change and pollution, and stricter emission laws, new approaches are being sought to produce novel and better quality products to meet the new sustainability targets of present politic and industrial thinking [1–4]. At this point, forest and mill residues, agriculture crops and wastes, wood and wood wastes, animal wastes, livestock operation residues, aquatic plants, fast-growing trees and plants, and municipal and industrial wastes can play an important role. These renewable resources can be transformed into a variety of products, including chemicals, energy, transportation fuels, and materials, giving rise of the concept of biorefinery as an integral unit which accepts, converts and processes such feedstocks [5–8].

For the sustainability of biorefineries, low environmental impact technologies must be used throughout, which may be accomplished by integrating that target into a performance criterion of the design. It is vital to avoid the unacceptable aspects of petrochemical production and avoid hazardous and environmentally harmful process auxiliaries, such as toxic reagents and volatile organic solvents. In this scenario, green chemical technologies, such as supercritical fluid extraction (SFE) [5,9], receive particular attention, and novel solvents become highly desirable, such as supercritical carbon dioxide (SC-CO_2_) and ionic liquids [1,4,10,11].

The interests of agro-forest industries, particularly pulp and paper mills, in the integrated exploitation of plants biomass is growing enormously, since they produce considerable amounts of by-products, such as bark and general logging wastes (e.g., leaves, branches, fruits, sawdust), which are either left in the forest for soil nutrition or ultimately burned in the biomass boiler for energy production. Some of these residues contain high value components whose extraction does not affect the current pulp and power outputs of existing mills. For instance, the exploitation of valuable extractives, such as phytosterols, namely *β*-sitosterol [12–14], lignans [15–17], and betulin [18,19] from by-products of the industrial processing (e.g., bark, wood knots, pulping liquors) is a strategy implemented in some pulp industries.

**Figure 1:**
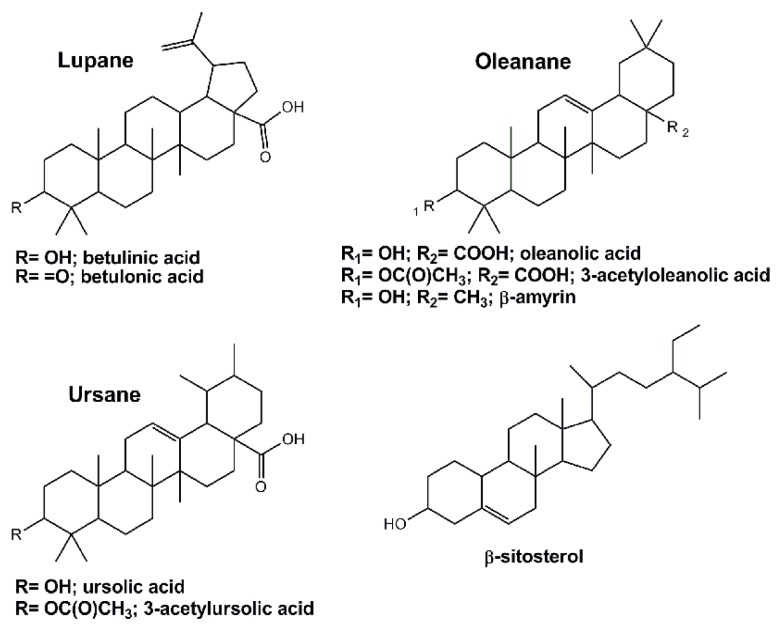
Major lupane, oleanane and ursane triterpenoids identified in *Eucalyptus globulus* bark.

In the last few years, we have been investigating the potential of *Eucalyptus spp.* residues in order to enlarge the concept “from waste to value” for pulp and paper mills [20–26]. Bark, particularly its outer fraction, is among the most interesting eucalypt residues, since it contains high amounts of triterpenoids, representing 5.2, 5.7, 9.3, 22.8 and 24.6 g/kg in *Eucalyptus urograndis*, *Eucalyptus grandis*, *Eucalyptus maidenii*, *Eucalyptus globulus* and *Eucalyptus nitens* respectively [20,22,23], along with monoterpenes and sesquiterpenes, followed by smaller amounts of fatty acids, fatty alcohols, and aromatic compounds [20–23]. The main triterpenoids found in several *Eucalyptus* species have lupane, oleanane and ursane structures, as shown in Figure 1: five triterpenic acids (ursolic, betulinic, oleanolic, betulonic, 3-acetylursolic and 3-acetyloleanolic acids), *β*-amyrin, and the triterpenic type sterol *β*-sitosterol. These triterpenic acids are recognized, for example, as promising compounds for the development of new multi-targeting bioactive agents [27–30] of very high market value.

For these reasons, the triterpenoid fraction of eucalyptus bark can justify a “high-value low-volume” outputs approach [6] in the integrated pulp mill biorefineries of the future.

In this paper, the supercritical fluid extraction of triterpenoids from eucalyptus deciduous bark is presented. The effects of pressure, co-solvent (ethanol) content, and multistep operation are studied in order to enlighten subsequent investigations on the applicability of SFE for their selective and efficient production. The extracts obtained from one step and multistep operation with SC-CO_2_ and SC-CO_2_ modified with ethanol are analyzed. The individual cumulative extraction curves of the main families of compounds are also discussed in detail.

## 2. Results and Discussion

### 2.1. Soxhlet Extraction of *Eucalyptus globulus* Deciduous Bark

**Figure 2:**
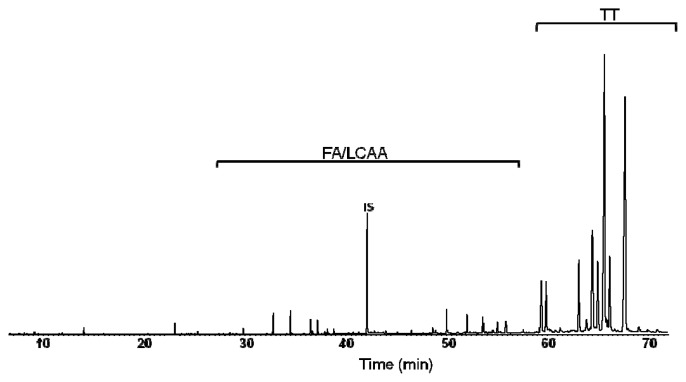
GC-MS chromatogram of the dichloromethane extract of *Eucalyptus globulus* deciduous bark. (FA, fatty acids; LCAA, long chain aliphatic alcohols; TT, triterpenoids; IS, internal standard-tetracosane).

The yield of the lipophilic extractives of deciduous bark extracted with dichloromethane was 2.1% (wt), which is in good agreement with previous results for this biomass fraction of *E. globulus* [21]. A GC-MS chromatogram of this extract (as TMS derivatives) is shown in Figure 2. In Table 1 the corresponding retention times, composition and concentrations are listed. It may be observed that the extract is mainly composed of several triterpenic acids with lupane, oleanane and ursane skeletons (Figure 1), mostly ursolic acid and its acetyl derivative, 3-acetylursolic acid, accounting for 2.77 g/kg and 2.64 g/kg, respectively, in a total of 10.74 g/kg quantified compounds. Betulonic (0.80 g/kg), oleanolic (0.71 g/kg), 3-acetyloleanolic (0.69 g/kg) and betulinic (0.62 g/kg) acids are also abundant in this extract, being the main components of the triterpenoid family of compounds, which also includes minor amounts of *β*-amyrin. Several fatty acids (C_14_ to C_28_, accounting globally for 0.48 g/kg), mainly hexadecanoic, tetracosanoic and hexacosanoic acids, some long chain aliphatic alcohols (C_16_ to C_28_, accounting globally for 0.44 g/kg), as hexacosan-1-ol, and *β*-sitosterol (0.24 g/kg) are also present in considerable amounts in the extract.

### 2.2. One-Step Supercritical Fluid Extraction of *Eucalyptus globulus* Deciduous Bark

**Figure 3:**
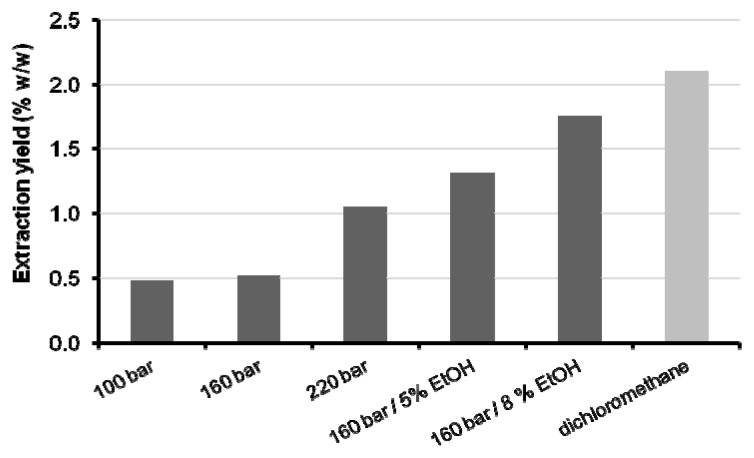
Global extraction yields of *Eucalyptus globulus* deciduous bark obtained at 40 °C with SC-CO_2_ and SC-CO_2_/ethanol compared to Soxhlet extraction with dichloromethane (EtOH, ethanol).

In this section, the results obtained for the five supercritical extractions carried out in one step are presented and discussed. On the whole, the extraction yields ranged from 0.48% to 1.76% (wt), a slight increase from 100 bar to 160 bar being observed (Figure 3), but a considerable jump of 103% was obtained when passing from 160 bar to 220 bar. This behavior is due to the direct effect of pressure upon the density of CO_2_, which determines solubility at constant temperature.

The densities of CO_2_, calculated with the Pitzer and Schreiber equation of state [31], for pressures of 100, 160, and 220 bar, are 633.1, 796.8 and 858.7 kg/m^3^, respectively, which shows the significant impact of density in the region around 800 kg/m^3^, a value typical of current organic solvents. An analogous trend was found for the extractions carried out at constant temperature and pressure (40 °C, 160 bar) when the co-solvent (ethanol) introduced was increased from 0% to 5%, and then to 8% (wt) (Figure 3). In this sequence, the densities of the CO_2_ and ethanol mixtures are very similar: 796.8, 794.2 and 795.0 [32]—but the extraction yields show two large increments (154% and 240%). Concerning the entrainer effect, which is defined as an increase in both solubility and selectivity [33], the polarity modification imparted by ethanol to the non-polar CO_2_, with final positive effect on solvent power, reveals the intermolecular interactions between ethanol and the extract components. It is worth noting that the yield achieved at 160 bar/40 °C/8% (wt) ethanol is close to that obtained by Soxhlet extraction with dichloromethane (1.8% *versus* 2.1%).

**Figure 4:**
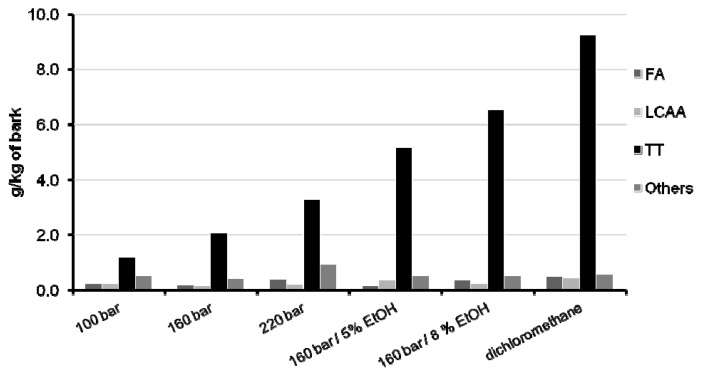
Contents of the main families of compounds in extracts of *Eucalyptus globulus* deciduous bark obtained with SC-CO_2_ and SC-CO_2_/ethanol at 40 °C (see Table 2). Comparison with Soxhlet extraction with dichloromethane. (FA, fatty acids; LCAA, long chain aliphatic alcohols; TT, triterpenoids; EtOH, ethanol).

Regarding the composition of those extracts, they exhibit considerable differences, both quantitatively and qualitatively. In Figure 4, the abundances of the most important chemical families detected are plotted together with the Soxhlet extraction results for comparison. The numerical values are listed in Table 2, where the individual concentrations of triterpenoids are specified, given their interest in this work.

In the assays without co-solvent addition, the extracts show similar compositions. The main components are triterpenoids, particularly 3-acetylursolic, 3-acetyloleanolic and betulonic acids, also as *β*-amyrin and *β*-sitosterol, followed by fatty acids, (from which palmitic and oleic are the most representative), and minor fractions of long chain aliphatic alcohols. *β*-sitosterol was included in the triterpenoids group. Besides, considering the recognized biological activities of this molecule [34,35], its eventual exploitation can be of relevant interest for the upgrade of *E. globulus* biomass residues. In comparison to the dichloromethane extract, it is noteworthy that the three assays without co-solvent have low content levels (or even an absence of content) of ursolic and oleanolic acids (Table 2), two of the main components of *E. globulus* deciduous bark (see Table 1). This is due to the polarity gap between them and CO_2_, which implies that higher pressure or, alternatively, a modifier is necessary to improve their solubility. This is clear from the successful removal of their acetylated forms (*i.e.*, 3-acetylursolic, 3-acetyloleanolic), since after esterification the polarity imparted by the hydroxyl group is much attenuated.

In experiments with co-solvent addition, a significant increase (151%) of the amount of triterpenoids extracted with 5% (wt) ethanol (5.17 g/kg at 160 bar) was observed in comparison to the extract obtained with pure carbon dioxide at the same pressure and temperature (2.06 g/kg)—see Table 2. The recovery of triterpenoids was once again enhanced (in this case, 26%) after raising the ethanol content to 8% (wt), attaining 6.53 g/kg of bark. These results emphasize the chief role played by solvent polarity, since such increments can be attributed mainly to the extraction of non-acetylated triterpenic acids. In fact, at 160 bar and 40 °C, their evolution along with ethanol percentage in the supercritical solvent may be taken from Table 2: ursolic acid, 0.073, 1.74, 1.78 g/kg, and oleanolic acid, 0.050, 0.66, 0.69 g/kg. In conjunction, these variations account for 52.5% of the global increment of triterpenoids. In contrast, the two acetylated acids (acetylursolic and acetyloleanolic), justify only 18.6% of triterpenoid extraction enhancement.

Compared with the dichloromethane extract, the quantities of triterpenoids obtained by SC-CO_2_/ethanol extraction reached about 70.7% of its total potential. Nonetheless, such yields may/should be optimized by adjusting operating conditions, such as extraction time, pressure, temperature, and co-solvent percentage in the SC-CO_2_ stream.

On the whole, the increased amount of triterpenoids extracted is, essentially, the main difference between the extracts obtained with and without co-solvent, the remaining composition being similar.

### 2.3. Stepwise SC-CO_2_ Extraction of *Eucalyptus globulus* Deciduous Bark

**Figure 5:**
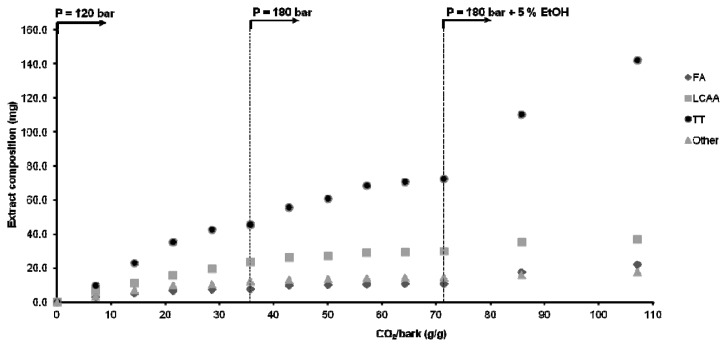
Cumulative extraction curves for each family of compounds obtained by stepwise extraction with SC-CO_2_ and SC-CO_2_/ethanol at 40 °C. (FA, fatty acids; LCAA, long chain aliphatic alcohols; TT, triterpenoids; EtOH, ethanol).

**Figure 6:**
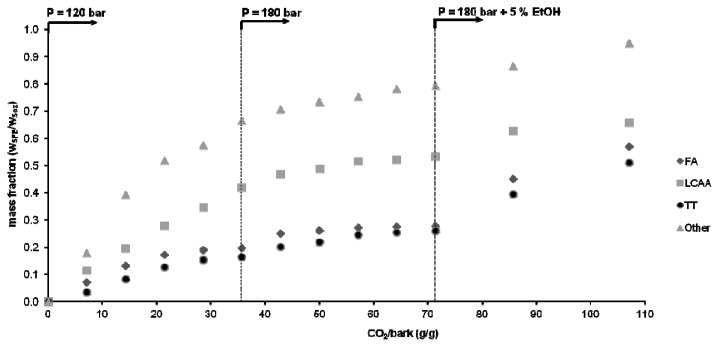
Cumulative extraction curves of Figure 5 normalized by the corresponding contents obtained by Soxhlet extraction with dichloromethane taken as reference values. (FA, fatty acids; LCAA, long chain aliphatic alcohols; TT, triterpenoids).

The individual cumulative curves for each family of compounds obtained in the stepwise extraction assay are plotted in Figures 5 and 6 as functions of the mass of the CO_2_ spent per unit mass of bark. In Figure 5, they are plotted as absolute weight (mg) of solute in the extract, while in Figure 6 they are normalized by their maximum extractable values (taken to be equal to that of the Soxhlet dichloromethane extract).

In the first step (120 bar, 40 °C), one extracts mostly long chain aliphatic alcohols and other compounds (41% and 66% of its total potential in the bark, respectively) and low quantities of triterpenoids and fatty acids. Of the triterpenoids, 3-acetylursolic acid is the predominant component due to its higher lipophilic character, at least in comparison with parent ursolic acid. Moreover, it should be noted that it represents *ca.* 30% of the triterpenoids extracted by Soxhlet with dichloromethane (see Table 1).

In the second step (180 bar, 40 °C), the improved solvent power of SC-CO_2_ explains the extraction of higher amounts of triterpenoids, which increased from *ca.* 55 to 72 mg (see Figure 5). It is worth noting that the extraction rate of triterpenoids and fatty acids increases instantaneously at the beginning of this step (see Figure 6 also), and then diminishes continuously with time, as in the first step. The remaining families of components do not exhibit this trend. That jump is essentially justified by the effect of pressure upon solubility, as already observed and discussed in section 2.2, whereas the deceleration is due to the fact that the driving force to mass transfer attenuates along the extraction. At higher pressures the proximity between CO_2_ and solute molecules is shortened, making possible interactions with CO_2_ quadrupole that are almost absent at low densities. Increasing density leads to the creation of substantial dipole (induced or not)–quadrupole interactions that favor the solubility of non-acetylated triterpenic acids, from which the ursolic acid must be detached because it is the most abundant (*ca.* 68%; see Table 1). As has been mentioned above, this enhancement can be largely incremented using ethanol as co-solvent, which has been accomplished in the following step.

Figure 6 illustrates that the introduction of ethanol (5%, wt) in step 3 contributed to the removal of higher amounts of triterpenoids and fatty acids, which doubled from 72 to 142 mg and from 10.8 to 22.2 mg, respectively, the variation of other families being much smaller. Nevertheless, this figure points out that we are still far from exhausting the total amount of triterpenoids present in eucalyptus deciduous bark; therefore an optimization work on this topic is still needed to improve the extraction yield. This will be the main target of future researches, in order to achieve selective and quantitative triterpenoid extraction using supercritical CO_2_ (modified or not) at economically tractable pressures.

Considering the results of this essay, one may expect that a multistep process based on different operating conditions may generate extracts enriched in triterpenoids by firstly removing part of the aliphatic matrix (fatty acids, long chain aliphatic alcohols and other compounds) with supercritical CO_2_, since numerous substances are CO_2_-philic.

## 3. Experimental Section

### 3.1. Chemicals

Nonacosan-1-ol (98% purity) and *β*-sitosterol (99% purity) were purchased from Fluka Chemie (Madrid, Spain); ursolic acid (98% purity), betulinic acid (98% purity) and oleanolic acid (98% purity) were purchased from Aktin Chemicals (Chengdu, China); betulonic acid (95% purity) was purchased from CHEMOS GmbH (Regenstauf, Germany); palmitic acid (99% purity), dichloromethane (99% purity), pyridine (99% purity), bis(trimethylsilyl)trifluoroacetamide (99% purity), trimethylchlorosilane (99% purity), and tetracosane (99% purity) were supplied by Sigma Chemical Co (Madrid, Spain). Carbon dioxide was supplied with a purity of 99.95% from Praxair (Porto, Portugal).

### 3.2. Bark Samples

Deciduous bark of *E. globulus* was randomly harvested from a 20-year-old clone plantation cultivated in the Eixo (40°37′13.56″N, 8°34′08.43″W) region of Aveiro, Portugal, air dried until a constant weight was achieved, and milled to granulometry lower than 2 mm prior to extraction. Deciduous bark was selected as substrate, since it is mostly outer bark (very similar to the last one in terms of triterpenic acids composition) and avoids felling a large number of trees to ensure a continuous supply of raw material of controlled origin for a long-term study.

### 3.3. Soxhlet Extraction

Samples of *E. globulus* deciduous bark (15 g) were Soxhlet-extracted with dichloromethane for 7 h. The solvent was evaporated to dryness, the extracts were weighed and the results were expressed as a percent of dry bark. Dichloromethane was chosen because it is a fairly specific solvent for lipophilic extractives and was used as a reference to evaluate the efficiency of the SFE extractions.

### 3.4. Supercritical Fluid Extraction (SFE)

#### 3.4.1. SFE Apparatus

**Figure 7:**
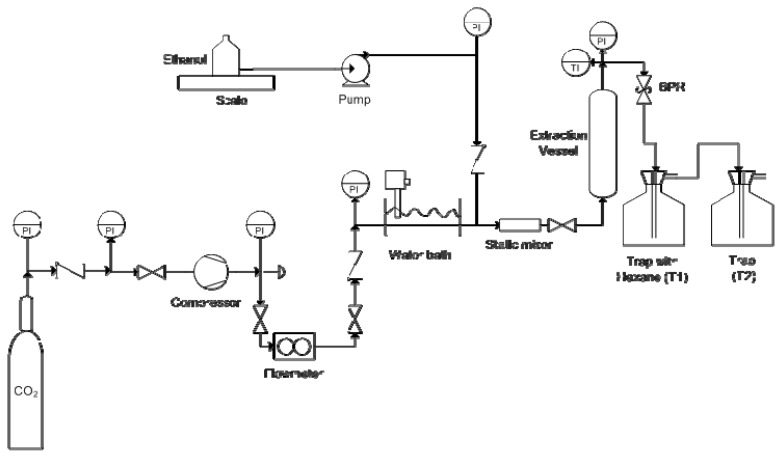
Flow diagram of the apparatus used for the supercritical fluid extraction experiments.

The SFE apparatus used to carry out the extraction assays is schematically shown in Figure 7. In this diagram the CO_2_ taken from a cylinder is compressed to the desired extraction pressure by means of a Nova Swiss gas compressor (model 5542121), and then heated to the desired temperature by passing through a high pressure tubing coil immersed in a temperature-controlled (up to ±0.5 °C) water bath. SC-CO_2_ flows at the desired pressure and temperature conditions upwards through a packed bed of *E. globulus* deciduous bark contained in the extraction vessel (316SS; internal diameter of 24 mm; total length of 572 mm). The extractor is heated by passing hot water through a heating jacket surrounding the outer surface of the vessel. The extraction pressure was controlled by means of a back pressure regulator, BPR (Tescom 27–1700), where depressurization of the extract flow stream took place. The extracts were solubilized in n-hexane and collected in a glass trap, T1. To ensure total recovery of compounds, the gas flow passes through a second glass trap, T2. Both traps are kept under 0 °C immersed in an ethylene glycol bath. The gas flow rate and total mass of carbon dioxide used in the assays are measured with a coriolis-type gas flow meter (Danfoss, Mass 6000). The extraction pressure is measured at the exit of the extraction vessel with an accuracy of ±0.1 MPa (Wika, model 881.14.600). Spent CO_2_ is vented to the atmosphere. The addition of co-solvent to the system was made by a liquid pump (LDC Analytical miniPump) coupled to the gas line between the high pressure tubing coil immersed in the water bath and the extraction vessel. SC-CO_2_ and the liquid co-solvent are mixed in a static mixer before entering in the extraction vessel. The co-solvent container is placed on a balance being the flow rate measured by weight difference and controlled by the liquid pump.

#### 3.4.2. SFE Procedure

In each run, about 30 g of milled bark were introduced in the extraction vessel. A first set of extractions were carried out at 100, 160 and 220 bar without the addition of co-solvent, and a second one at 160 bar with 5% and 8% (wt) ethanol during 3 h. The average SC-CO_2_ flow rate was 12.5 g/min, and the temperature was kept at 40 °C. The extracts were collected in both traps at the end of each run and combined.

A second sequence of extractions in series was performed at 40 °C using about 70 g of milled bark and 6 g/min of solvent. The following steps of 5 h were carried out: (1) extraction at 120 bar with pure CO_2_; (2) extraction at 180 bar with pure CO_2_; and (3) extraction at 180 bar with CO_2_ modified with 5% ethanol. The extracts were collected within 1 h interval in the first two steps, while in step 3 they were collected after 2 and 5 h of extraction time. All extracts were collected from both traps, combined and analyzed individually. The solvent was evaporated to dryness. The extracts were weighed and the results expressed as a percentage of dry bark.

### 3.5. GC-MS Analyses

Before each GC-MS analysis, nearly 20 mg of dried sample were converted into trimethylsilyl (TMS) derivatives according to the literature [23]. GC-MS analyses were performed using a Trace Gas Chromatograph 2000 Series equipped with a Thermo Scientific DSQ II mass spectrometer, using helium as carrier gas (35 cm·s^−1^), equipped with a DB-1 J&W capillary column (30 m × 0.32 mm i.d., 0.25 μm film thickness). The chromatographic conditions were as follows: initial temperature: 80 °C for 5 min; temperature rate of 4 °C·min^−1^ up to 260 °C and 2 °C min^−1^ until the final temperature of 285 °C; maintained at 285 °C for 10 min; injector temperature: 250 °C; transfer-line temperature: 290 °C; split ratio: 1:50. The MS was operated in the electron impact mode with electron impact energy of 70 eV and data collected at a rate of 1 scan·s^−1^ over a range of *m/z* 33–700. The ion source was maintained at 250 °C.

For quantitative analysis, the GC-MS instrument was calibrated with pure reference compounds, representative of the major lipophilic extractives components (namely, palmitic acid, nonacosan-1-ol, *β*-sitosterol, betulinic acid, ursolic acid and oleanolic acid), relative to tetracosane, the internal standard used. The respective multiplication factors needed to obtain correct quantification of the peak areas were calculated as an average of six GC-MS runs. Compounds were identified, as TMS derivatives, by comparing their mass spectra with the GC-MS spectral library, with data from the literature [23,36–41] and, in some cases, by injection of standards. Two aliquots of each extract were analyzed. Each aliquot was injected in triplicate. The results presented are the average of the concordant values obtained for each part (less than 5% variation between injections of the same aliquot and between aliquots of the same sample).

## 4. Conclusions

In this work, eucalyptus deciduous bark was investigated as a source of triterpenoids due to their interest as new bioactive agents. The main components are ursolic acid and its acetyl derivative, 3- acetylursolic acid, which accounted for 2.77 and 2.64 g/kg, respectively, in a total of 10.74 g/kg of quantified compounds. The supercritical fluid extraction with pure and modified carbon dioxide was evaluated by carrying out experiments at 40 °C at pressures from 100 to 220 bar. Pressure has a large influence upon the extraction yield and on the concentrations of the extracts. Furthermore, the introduction of 8% (wt) of ethanol at 160 bar and 40 °C more than trebles the yield of triterpenoids, which highlights the important role played by co-solvent in this extraction. Hence, ethanol may be used with advantage, since its effect is more important than increasing pressure by several tens of bar. Taking into account the additional multistep extraction performed in series in this work, the results showed that an appropriate combination of operating conditions may generate extracts enriched in triterpenoids.

## Figures and Tables

**Table 1:** Composition of the dichloromethane extract of *Eucalyptus globulus* deciduous bark. The retention times correspond to the chromatogram of Figure 2.

*t_r_* (min)	Compound	Content (mg/kg of bark)
	**Fatty acids (FA)**	**482.8**
29.81	tetradecanoic acid	40.6
34.50	hexadecanoic acid	109.2
38.15	linoleic acid	16.7
38.82	oleic acid	22.3
42.10	octadecanoic acid	21.6
48.61	docosanoic acid	27.6
50.15	tetracosanoic acid	114.0
55.03	hexacosanoic acid	109.3
59.87	octacosanoic acid	21.6

	**Long chain aliphatic alcohols (LCAA)**	**445.5**
32.73	hexadecan-1-ol	96.6
36.49	*Z*-9-octadecen-1-ol	81.9
37.19	*E*-9-octadecen-1-ol	23.6
37.94	octadecan-1-ol	62.6
49.99	tetracosan-1-ol	25.6
53.58	hexacosan-1-ol	85.9
59.37	octacosan-1-ol	69.1

	**Sterols (ST)**	**242.5**
60.13	*β-*sitosterol	242.5

	**Triterpenoids (TT)**	**9,001.7**
60.07	*β-*amyrin	313.7
64.21	betulonic acid	797.7
64.96	oleanolic acid	712.5
65.86	betulinic acid	618.7
66.18	ursolic acid	2,771.9
66.68	3-acetyloleanolic acid	691.2
66.97	3-acetylbetulinic acid	55.1
68.03	3-acetylursolic acid	2,635.1
	unidentified triterpenoids	405.8

	**Other compounds/unidentified compounds**	**566.5**
	**Total detected compounds**	**10,738.9**

**Table 2:** Main components and families (mg/kg of dry bark) of *Eucalyptus globulus* deciduous bark extracts obtained with SC-CO_2_ and SC-CO_2_/ethanol. Comparison with Soxhlet results using dichloromethane (see Table 1).

	Supercritical Fluid Extraction b					Soxhlet

P (bar)	100	160	220	160	160	Dichloromethane 7 h

Ethanol (%, wt)	0	0	0	5	8	
**Fatty acids**	**243.8**	**189.5**	**374.8**	**174.8**	**351.9**	**482.8**
**Long chain aliphatic alcohols**	**246.2**	**173.7**	**226.5**	**345.3**	**253.1**	**445.5**
**Triterpenoids**	**1,193.4**	**2,063.3**	**3,273.0**	**5,174.4**	**6,534.1**	**9,244.2**
*β*-sitosterol	190.6	131.1	236.8	272.0	261.5	242.5
*β*-amyrin	137.6	91.8	171.7	151.1	168.6	313.7
betulonic acid	198.6	399.8	648.1	653.8	731.6	797.7
oleanolic acid	ND a	49.9	79.5	661.1	691.4	712.5
betulinic acid	ND a	123.7	177.5	572.4	633.0	618.7
ursolic acid	ND a	73.0	69.4	1,740.5	1,779.6	2,771.9
3-acetyloleanolic acid	143.9	238.5	384.6	172.0	429.1	691.2
3-acetylursolic acid	409.9	875.6	1,377.6	820.6	1,495.3	2,635.1
Other/unidentified triterpenoids	112.8	79.9	127.8	130.9	344.0	460.9
**Other compounds**	**520.4**	**398.3**	**926.2**	**523.6**	**521.9**	**566.5**
**Total detected**	**2,203.8**	**2,824.8**	**4,800.5**	**6,218.2**	**7,661.0**	**10,739.0**

a ND = not detected;

b Remaining SFE conditions: extraction temperature, 40 °C; CO_2_ mass flow rate, 12.5 g/min; extraction time, 3 h.
